# Impact of Nanoparticle Addition on the Surface and Color Properties of Three-Dimensional (3D) Printed Polymer-Based Provisional Restorations

**DOI:** 10.3390/nano14080665

**Published:** 2024-04-11

**Authors:** Maram A. AlGhamdi, Fatimah M. Alatiyyah, Rawan F. Almedarham, Zainab H. Al Dawood, Farah Y. Alshaikhnasser, Shaymaa Y. Alboryh, Soban Q. Khan, Reem Abualsaud, Mohammed M. Gad

**Affiliations:** 1 Department of Substitutive Dental Sciences, College of Dentistry, Imam Abdulrahman Bin Faisal University, P.O. Box 1982, Dammam 31441, Saudi Arabia; maalghamdi@iau.edu.sa (M.A.A.); rabualsaud@iau.edu.sa (R.A.); 2 College of Dentistry, Imam Abdulrahman Bin Faisal University, P.O. Box 1982, Dammam 31441, Saudi Arabia; 2180006396@iau.edu.sa (F.M.A.); 2180004025@iau.edu.sa (R.F.A.); 2180003167@iau.edu.sa (Z.H.A.D.); 2180004807@iau.edu.sa (F.Y.A.); 2180002447@iau.edu.sa (S.Y.A.); 3 Department of Dental Education, College of Dentistry, Imam Abdulrahman Bin Faisal University, P.O. Box 1982, Dammam 31441, Saudi Arabia; sqkhan@iau.edu.sa

**Keywords:** 3D printing, CAD-CAM, provisional resins, nanoparticles, surface properties, optical properties

## Abstract

This study aimed to evaluate and compare the impact of additives such as ZrO_2_ and SiO_2_ nanoparticles (ZrO_2_NP or SiO_2_NP) on the hardness, surface roughness, and color stability of 3D printed provisional restorations. Two hundred samples in total were printed using 3D printed resins (ASIGA, and NextDent). Each resin was modified with ZrO_2_NPs or SiO_2_NPs in two different concentrations (0.5 wt% and 1 wt%), while one group was kept unmodified (n = 10). Disc-shaped (15 × 2.5 mm) samples were designed and printed in accordance with the manufacturer’s recommendation. Printed discs were evaluated for color changes through parameters CIELAB 2000 system (ΔE_00_), hardness using Vickers hardness test, and surface roughness (Ra) using a noncontact profilometer. After calculating the means and standard deviations, a three-way ANOVA and Tukey post hoc test were performed at α = 0.05. The addition of ZrO_2_NPs or SiO_2_NPs to ASIGA and NextDent resins significantly increased the hardness at a given level of concentration (0.5% or 1%) in comparison with pure (*p* < 0.001), with no significant difference between the two modified groups per resin type (*p* > 0.05). The highest hardness value was detected in 1% ZrO_2_NPs with 29.67 ± 2.3. The addition of ZrO_2_NPs or SiO_2_NPs had no effect on the Ra (*p* > 0.05), with 1% ZrO_2_NPs showing the highest value 0.36 ± 0.04 µm with NextDent resin. ZrO_2_NPs induced higher color changes (∆E_00_), ranging from 4.1 to 5.8, while SiO_2_NPs showed lower values, ranging from 1.01 to 1.85, and the highest mean ∆E_00_ was observed in the 1% ZrO_2_NPs group and NextDent resin. The incorporation of ZrO_2_NPs and SiO_2_NPs in 3D printed provisional resins increased the hardness without affecting the surface roughness. The optical parameters were significantly affected by ZrO_2_NPs and less adversely affected by SiO_2_NPs. Consequently, care must be taken to choose a concentration that will improve the materials’ mechanical performance without detracting from their esthetic value.

## 1. Introduction

Provisional restorations are tooth-shaped shells that are used for a limited period until they are replaced by a definitive prosthesis [1]. They are often referred to as temporary restorations and they are made to temporarily protect and maintain the mechanical and biological integrity of the prepared teeth and the surrounding tissues during the treatment [2,3]. They safeguard the prepared tooth from thermal insults, provide stable occlusal contacts, and help in assessing the ultimate treatment’s therapeutic, functional, and cosmetic efficacy [3,4].

Three-dimensional (3D) printing technologies have opened many possibilities in a variety of areas, including healthcare [5]. A key element of this technology is 3D printed resins, used for provisional restorations, which have distinct advantages [5]. When it comes to making complicated geometry, traditional manufacturing processes frequently have restrictions, but 3D printing allows for detailed designs that may be adjusted to individual demands [5,6]. Furthermore, resins offer a wide range of qualities, including flexibility, strength, and transparency [6,7]. This adaptability permits the production of objects with a vast range of functions and applications [6,8]. However, there are also disadvantages associated with 3D printed resins. One of the major drawbacks is the limited range of available materials and their associated mechanical properties [8,9]. While this technology is continuously evolving, the variety of materials currently offered is lower compared to that of the conventional manufacturing techniques [8]. This restriction may limit certain applications and industries that require specific material properties [5].

Provisional restorations serve as placeholders until the permanent dental prosthetics’ placement, offering patients both functional and esthetic benefits during the interim period [2,3,10]. Commonly utilized provisional materials include polymethyl methacrylate (PMMA) resins, bis-acryl composites, and light-cured materials [4]. The most popular provisional restorations, known as (PMMA), are composed of prepared synthetic polymer [8]. At the moment, these are the materials that are most frequently used. The enhanced (Bis-GMA) material later made its way into various industries, and Bis-acryl resins are now widely used in the production of provisional fixed prostheses [2,3]. The selection of a suitable provisional material is dependent on many factors, including the intended duration of use, esthetic demands, and the clinical case scenario [10]. The issue of creating heat during the process of polymerization is a concern shared by dentists when using all provisional resins [11,12]. Since the hardness and yield strength diminish with increasing temperature, abrasive wear would increase [12]. Temperature-related decreases in yield strength and hardness occur in most materials. When tribological and dynamic mechanical analysis were used in the problem-solving process, the researchers discovered that using nano liquid reduced the amount of heat produced during wear and tear when compared to standard fluid [11,12]. The fillers or fibers that are added to polymers to help reinforce the surface were shown to improve the polymer’s tribological performance. This essentially covers the loading parameters for adhesive wear in dry contact situations [11,12].

Moreover, advanced manufacturing techniques have fundamentally impacted the development of provisional materials, enabling the creation of more precise and durable provisional restorations [7,8]. Computer-aided design and computer-aided manufacturing (CAD/CAM) technologies, including 3D printing, have revolutionized production, enhancing the accuracy and efficiency of provisional restorations [8,9]. These advancements contribute to the overall success of dental treatments by providing patients with well-fitted and esthetically pleasing temporary solutions.

A high-quality definitive fixed dental prosthesis (FDP) depends on a well-made provisional restoration [13,14]. The function and esthetics of a provisional FDP must be established while preserving the periodontal relationship and the alignment of the tooth [13]. The materials’ composition and manufacturing method influence the properties of 3D printed provisional restoration [14,15]. Comparing the physical properties of 3D printed provisional resins to conventional and subtractive techniques to fabricate provisional materials revealed inferior results. The main cause of poor mechanical properties, such as low hardness values in 3D printed materials, was reported to be due to shrinkage during the layering and post-processing steps [16,17]. Physical properties like increased surface roughness in 3D printed materials resulted in rapid material discoloration that intensified as time elapsed, resulting in a perceptible color change (ΔE) [15]. In order to enhance these qualities for long-term provisionalization, modifications such as the addition of different ingredients to 3D printed provisional resin may be attempted.

Nanoparticles have emerged as a promising method to address drawbacks associated with conventional provisional resin materials [17]. Integrating nanoparticles, particularly those composed of zirconia and silica, into provisional resin provides improved mechanical properties, enhanced wear resistance, and increased stability [15,18,19]. Zirconia nanoparticles, known for being non-toxic, holding high mechanical-physical properties, and having high strength and toughness, contribute to the enhancement of provisional resin, minimizing the risk of fracture and reinforcing the overall durability. These are added to improve strength by reducing the porosity and surface roughness, and increasing the hardness [20,21]. Previous investigations evaluating the mechanical effects of zinc oxide particles found that NP-modified dental-restorative-composite materials showed improved properties [22]. Silica nanoparticles, on the other hand, aid in achieving a smoother and more polished surface, improving the esthetic qualities of provisional restorations [23,24]. The distinctive properties of these nanoparticles, such as their high surface area and reactivity, contribute to the modification of the material’s characteristics [25]. The controlled incorporation of zirconia and silica nanoparticles into provisional resins can result in materials with superior mechanical performance and esthetic outcomes [24,25]. This innovative approach showcases the potential of nanotechnology in overcoming the limitations associated with traditional provisional materials, paving the way for improved clinical success and patient satisfaction [21,22,23,24].

The color and surface properties of provisional resins are crucial aspects that significantly impact the esthetic outcome and patient satisfaction of a provisional dental restoration [25]. Provisional resins, often used as interim crowns or FPDs, should exhibit color stability, mimic natural teeth, and possess desirable surface characteristics to ensure harmonious integration with the patient’s dentition [2,3]. Any alteration in the provisional resin color over time can result in an unesthetic appearance [25,26]. Resin composition, and exposure to environmental factors may disturb the color of provisional resins [23]. Studies have investigated the degree of color change of various provisional materials and the possible advancements in resin technology in an attempt to improve their esthetic longevity [15,16,23]. Surface properties, including roughness and polishability, play an important role in patient comfort, hygiene, and satisfaction. A smooth and polished surface helps resist plaque accumulation, decreases pigmentation, enhances esthetics, and contributes to the overall longevity of the provisional restoration [15,16].

Based on these studies, the 3D printed nanocomposite provisional showed superior wear resistance compared to the available provisional restorations [15,16]. However, further investigations to assess the physical properties are needed. In the literature, no prior studies investigated and compared the concept of ZrO_2_NPs and SiO_2_NPs incorporation in different 3D printed provisional material. Therefore, this article’s objective is to explore the effects of ZrO_2_NPs and SiO_2_NPs addition on the hardness, roughness, and color change of 3D printed provisional resins. The null hypothesis stated that the addition of ZrO_2_NPs and SiO_2_NPs to 3D printed resin at varying concentrations will not have significant effects on the surface properties and color of 3D printed nanocomposite provisional resins.

## 2. Materials and Methods

### 2.1. Sample Size and Grouping According to Materials and Nanoparticle Type and Concentrations

A recent study [27] that assessed the properties of 3D printed provisional resin was used as a guide to calculate the sample size. A total of 200 (100 for hardness and 100 for roughness and color changes) specimens [50/resin, 10/pure, 20/0.5%, and 20/1% (10/ZrO_2_NP and 10/SiO_2_NPs)] were tested for hardness, surface roughness, and color change [28,29]. The power analysis was calculated using the WHO formula, with 80% study power, 5% level of significance, and a 5% marginal error.

### 2.2. Preparation of Nanocomposite

Two different NPs were surface-treated as described in Table 1, and each NP was added to a single bottle containing nanocomposite liquid resin. Nanocomposite mixtures were prepared with NPs/resin filling ratios by weight as follows: 0.5% group (0.5% NP/99.5% fluid resin) and 1% group (1% NP/99% fluid resin) resulted in total 100 g nanocomposite per group. The weight of the salinized NPs was determined using a digital scale (S-234; Denver Instrument, Gottingen, Germany), followed by mixing with two resin types [NextDent (Shade N1) and ASIGA (Shade A1)]. According to NPs type and concentration, 5 groups (n = 50) were prepared per resin; 1 pure with no additions (control), and 4 groups with NP additions (0.5% ZrO_2_NPs, 1% ZrO_2_NPs, 0.5% SiO_2_NPs, and 1% SiO_2_NPs).

### 2.3. Printing Parameters

**Figure 1:**
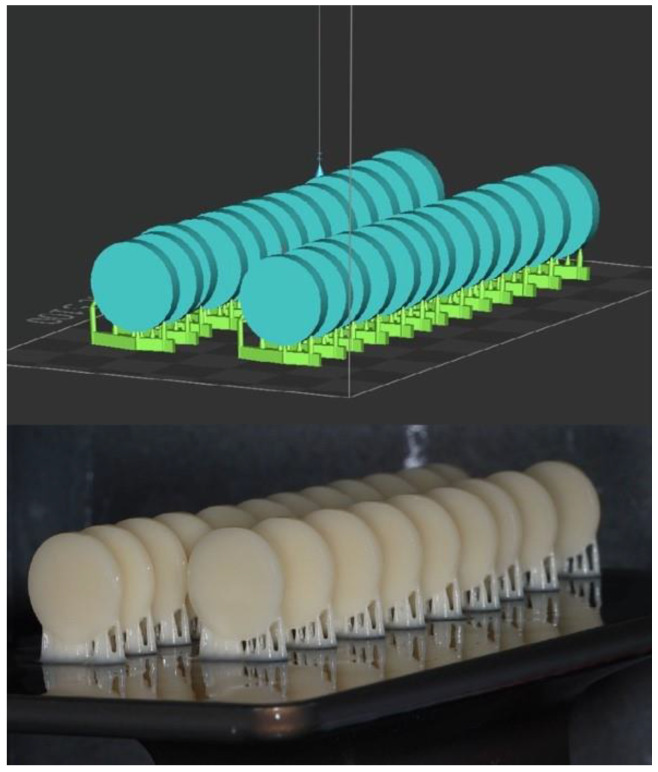
Specimens’ design (STL file) and printed specimens in printing plate.

A disc specimen with dimensions of 15 × 2 ± 0.2 mm was designed using CAD software (123D design, Autodesk, version 2.2.14, San Francisco, CA, USA) [27,28]. The file of the design was saved as a standard tessellation language (STL) file (Figure 1). Using the STL file, 100 discs were 3D printed using the corresponding 3D printer for each resin type as listed in Table 2 and shown in Figure 1. Samples were put into curing machines after being submerged in a glycerol-based bath for the post-curing time, which was set at 30 min at 80 °C, to obtain the final material characteristics. Afterwards, the 3D printed discs were trimmed from the lattice support with a diamond disc to obtain the final shape and remove unpolymerized resin [28,29]. Specimens were wet-ground using an automated polishing machine (EcoMetTM 30 Semi-Automatic Grinder Polisher, Buehler, IL, USA) with a standard grinding paper (grit range of 320–1200) to achieve smooth surfaces while maintaining the same dimensions [28,29]. The samples were immersed in 37 °C distilled water for 48 h prior to testing. Each laboratory step was conducted by a single investigator for both resin materials.

### 2.4. Testing Procedures

#### 2.4.1. Hardness

The discs were subjected to Vickers hardness indenter (HMV-2 Shimadzu Corp., Tokyo, Japan) [28,29] at 100 g of force for 15 s (ASTM C1327-03 standard [30]) [29,31] at three random locations on the specimen’s surface. The specimen’s hardness value (VH) was the average of the three readings.

#### 2.4.2. Surface Roughness (Ra, µm)

A noncontact profilometer (Contour GT-K 3D Optical Microscope, Bruker, Billerica, MA, USA) was utilized to determine the surface roughness. The Ra value of each specimen was determined by averaging the values of 3 readings at different locations on the surface [32,33].

#### 2.4.3. Color Stability

Color measurements were obtained with a standard illuminant C and calibrated spectrophotometer (Color-Eye^®^ 7000 A, X-Rite, Carlstadt, NJ, USA) in the visible spectrum range (380–780 nm) [32,34]. The color difference between the specimens was calculated using the following equation:

$$\Delta E_{00}=\sqrt{{\left(\frac{\Delta L^{\prime }}{K_{L}S_{L}}\right)}^{2}+{\left(\frac{\Delta C^{\prime }}{K_{C}S_{C}}\right)}^{2}+{\left(\frac{\Delta H^{\prime }}{K_{H}S_{H}}\right)}^{2}+{\;R}_{T}\left(\frac{\Delta C^{\prime }}{K_{C}S_{C}}\right)\;\left(\frac{\Delta H^{\prime }}{K_{H}S_{H}}\right)}$$

where ΔL′, ΔC′, and ΔH′ are the differences in lightness, chroma, and hue between the control and modified specimens, R_T_ is a function that accounts for differences in chroma and hue in the blue region, and S_L_, S_C_, S_H_ adjust for variation in the location of color difference of samples in L′, a′, b′ values, while K_L_, K_C_, K_H_ are correction terms set at 2:1:1 [15,32,33]. ΔE_00_ was assessed on perceptibility (PT) basis and acceptability (AT) thresholds, which were set at 50:50%; PT ranged from 0.8 to 1.30 and 50:50% AT ranged from 1.80 to 2.25 ΔE_00_ [35].

### 2.5. Data Analysis

The statistical package for social sciences (SPSS v.23) was used for statistical analysis. Means and standard deviations were calculated. The insignificant results of the Shapiro–Wilk test suggested a normal distribution of the data. Therefore, parametric tests were used for the inferential analysis. One-way ANOVA was used to study the difference between the means of the groups followed by Tuckey post hoc test for the pair-wise comparisons where applicable. Three-way ANOVA was used to assess the effect of the interaction between factors (loading level, NP, and material) on the tested properties. *p*-values of less than 0.05 were considered statistically significant.

## 3. Results

Table 3 demonstrates the variation in the hardness caused by the NPs’ concentration levels. The ANOVA results showed significant differences between the groups for both resins (*p* ≤ 0.01). The addition of ZrO_2_NPs or SiO_2_NPs significantly increased the hardness in comparison with pure groups (*p* = 0.01, *p* < 0.001), respectively. For pairwise comparisons of modified groups, no significant differences between the two concentrations in both NPs and resin type were detected (*p* > 0.05). The collective effect of two and three factors was evaluated using three-way ANOVA and the results were tabulated in Table 4. It was found that none of the combined factors were statistically significant, whether two or three factors.

The means and standard deviations of Ra of tested groups are presented in Table 3. The ANOVA results showed no significant differences in Ra between the groups for both resins and both NPs (*p* > 0.05). While 1% SiO_2_NPs recorded the highest Ra value (0.32 ± 0.09 µm) between the ASIGA groups, 1% ZrO_2_NPs showed the highest Ra value (0.36 ± 0.04 µm) for NextDent. Generally, NextDent showed higher values in comparison to ASIGA for all NP concentrations. The ANOVA results for surface roughness are shown in Table 3. The results indicated that only the combined effect of NP × material had a significant effect on the surface roughness (*p* = 0.045). Otherwise, none of the factors (two or three) were statistically significant.

**Figure 2:**
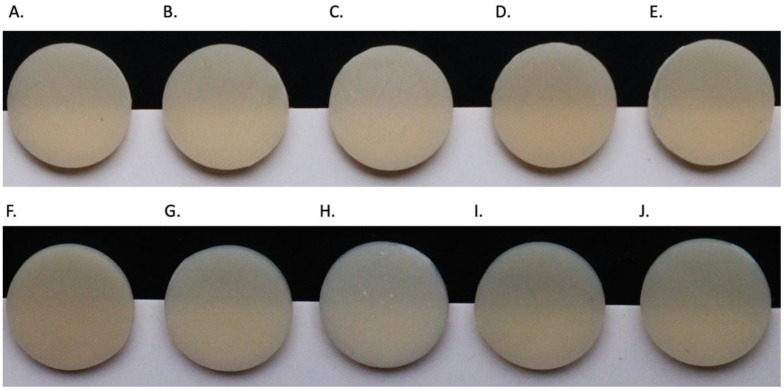
Photographs of the samples (**A**) ASIGA (pure), (**B**) ASIGA (0.5% ZrO_2_NPs), (**C**) ASIGA (1% ZrO_2_NPs), (**D**) ASIGA (0.5% SiO_2_NPs), (**E**) ASIGA (1% SiO_2_NPs), (**F**) NextDent (pure), (**G**) NextDent (0.5% ZrO_2_NPs), (**H**) NextDent (1% ZrO_2_NPs), (**I**) NextDent (0.5% SiO_2_NPs), and (**J**) NextDent (1% SiO_2_NPs).

Table 4 presents the color change results (ΔE_00_) for resins with different percentages of ZrO_2_NPs and SiO_2_NPs additions. Significant color changes were observed with ZrO_2_NPs, and this change was concentration dependent. In addition, 1% ZrO_2_NPs specifically showed a substantial increase in ΔE_00_ compared to 0.5% ZrO_2_NPs in both resins. Regarding SiO_2_NPs, no significant difference in ΔE_00_ was observed. When comparing the NPs, ZrO_2_NPs induced higher color changes (ΔE_00_) ranging between 4.1 ± 0.4 and 6.2 ± 0.6 in contrast to 1.01 ± 0.4 and 2.12 ± 0.4 for SiO_2_NPs. Also, the findings were supported by actual images of representative specimens per group (Figure 2). The combined effects of two and three factors on ΔE_00_ were studied using three-way ANOVA (Table 3). The results showed that the combined effect of NP × concentration (*p* = 0.000) and NP × material (*p* = 0.000) in addition to the effect of all three factors combined (*p* = 0.004) had significant effects on ∆E_00_.

## 4. Discussion

In a previous systematic review by Gad and Fouda, the elements influencing the mechanical properties of 3D printed resins were extensively explored [36]. Within the context of resin modifications, nanoparticles in 3D printed resins represent a noteworthy factor impacting their overall properties, as previously reported and classified [24,25,36]. This investigation assessed the hardness, surface roughness, and color change of 3D printed provisional resins modified with ZrO_2_NPs and SiO_2_NPs. This study’s results dictated a partial rejection of the null hypothesis, indicating significant differences in hardness and color change, while no statistically significant effect was observed on the roughness of 3D printed nanocomposite provisional resins.

Prior research has demonstrated that using nanoparticles as reinforcement can enhance the qualities of materials [17,27]. The advantageous effects of modified materials incorporating nanoparticles are limited by several criteria, including size, shape, ratio, homogeneous dispersion within the matrix of the resin, and salinization phase [17,27]. The filler’s weight percentages had an even more noticeable effect on the mechanical and physical characteristics [27]. To improve the chemical interaction between the filler phase and polymer matrix, the NPs’ surfaces were silanized [27]. The optical characteristics of the modified material would be improved by acceptable chemical bonding since the fillers and their quantity are important factors [31]. ZrO_2_NPs and SiO_2_NPs have been shown in earlier studies to improve the mechanical qualities of 3D printed materials [31]. Resin with high concentrations of ZrO_2_NPs is whitish in color, while SiO_2_NPs might limit light transmission and lower translucency [27,31]. Therefore, it is crucial to figure out the exact concentration of the filler to guarantee that it adheres to the resin matrix without forming clusters [34,37]. The use of NPs at high concentrations may have unfavorable effects resulting from the particles’ potential to cluster [34,37]. Previous studies reported that low quantities of SiO_2_NPs improved 3D printed resin, while 3 wt% ZrO_2_NPs resulted in maximum improved properties. On the other hand, concentrations above 7% were avoided due to the possibility of remarkable color change [27,31]. To compare the two nanoparticles and lessen the color change effect in terms of esthetic requirements with ZrO_2_NPs, 0.5 wt% and 1 wt% were introduced in the current study.

Hardness is likewise impacted by the ratio of organic to inorganic substance. It is used to determine a material’s ability to withstand abrasion, resist indentations, and abrade the opposing structure, and as an indirect way to determine the degree of polymerization [14,38,39,40]. This investigation revealed that the addition of ZrO_2_NPs and SiO_2_NPs increased the hardness at a given level of concentration (0.5% or 1%). This significance was found for NPs and both materials (NextDent and ASIGA). The results are compatible with the findings of Aati et al. [27], who found that the hardness of ZrO_2_NPs-modified 3D printed materials increased positively in relation to the filler weight increase [27]. In this study, the lowest and highest hardness values were 22.3 and 29.67, respectively. However, Aati et el., in a previous study, reported the highest hardness value of 20.02 [27]. The positive effects of ZrO_2_NPs in our study can be explained by their small diameter and ability to spread stresses within the resin more effectively [27]. Similarly, Gad et al. [41] discovered that, despite a barely noticeable variation in concentration, the hardness of 3D printed denture resin modified with SiO_2_NPs increased significantly [41]. In this study, disregarding the type of additive, microhardness increased in a direct relation with filler proportion. This increase might be linked to the advantages of the nanosized fillers used [27,42]. The nanosize of the particles and their homogenous dispersion within the material helped in reducing interparticle spacing and enhancing the microhardness of the modified nanocomposite, leading to higher capacity of indentation resistance [41,43]. The oxidative metals ZrO_2_NPs and SiO_2_NPs used in this study are considered to exhibit superior mechanical strength and hardness [27,42]. In contrast to pure resin, it is anticipated that various fractional enhancements of nanofillers would greatly improve the durability of 3D printed resin, with higher filler content translating into higher microhardness [41,43]. The nanofillers are predicted to significantly improve the mechanical properties due to high interfacial shear strength, which was increased by appropriate silanization and interphase interaction [41,44].

The physical and chemical characteristics of the 3D printed provisional material control how long the restorations last [43]. When fabricating restorations, surface roughness is a significant physical attribute that should be considered. Lack of esthetics, possibility of staining and loss of glossy appearance are strongly associated with rough surfaces [45,46]. Furthermore, a strong correlation has been demonstrated between roughness and other concerns like plaque accumulation, gingival inflammation, and caries [45,46,47]. Consequently, smooth surfaces are required. It was determined that different manufacturing processes may influence the material’s susceptibility to plaque adherence, and the established Ra threshold was 0.2 μm [47]. In this study, the Ra value range of pure specimens was between 0.26 µm and 0.33 µm, and the Ra values of tested samples varied between 0.23 µm and 0.36 µm. In addition, the statistical analysis revealed no significant change in surface roughness at all ZrO_2_NPs and SiO_2_NPs levels for both resins (NextDent and ASIGA). These findings are consistent with earlier research stating that the addition of ZrO_2_NPs had no effect on surface roughness [48]. Previously, investigations have added ZrO_2_NPs and SiO_2_NPs to a 3D printed provisional at 5% and 10% loading level and concluded that the incorporation increased the surface roughness values, especially 10%SiO_2_NPs [43]. In a different investigation, an interim prosthesis with loaded with 1% ZrO_2_NPs demonstrated no change in surface roughness compared to the control [45].

According to the literature, the influence of nanoparticles on surface roughness varied depending on the amount of nanofiller used [43,45]. The hardness of 3D printed resin was enhanced after the incorporation of 0.5% and 1% SiO_2_NPs, but Ra was not notably changed. This is in alignment with the fact presented in a previous study where the Ra of all 3D printed resin groups was significantly higher than that of the PMMA group (*p* < 0.001), with little variation observed among the 3D printed groups [41]. This noted increase in roughness with SiO_2_NPs loading compared to PMMA was attributed to cluster formations at the samples’ surfaces [41]. In contrast, loading 3D printed resin with ZrO_2_NPs did not affect the roughness owing to the filler’s homogenous distribution within the polymer matrix, which is less likely to affect the surface roughness [27,31,41]. A variety of factors affected the surface roughness, such as the nanoparticles’ tendency to cluster when the concentration rises, which frequently results in lower homogeneity of the composite resin [27,39,42]. During the polishing process, the superficial clustered particles disengage, creating voids and holes [44]. The printing technique (layer by layer and manufacturing direction) could be another cause of the alternation in Ra. When the sample surfaces are inclined or tilted in relation to the platform, the layered structure of the resin may create a stair-stepping effect [41]. In certain cases, the removal of the support structures leaves residues on the surface that degrade the surface properties. These effects are highly dependent on how a part surface is oriented during the building process. Also, surface properties reflect any errors or irregularities that might arise during the process of NPs’ mixing and printing [41]. Nevertheless, the surface roughness in the current investigation was not impacted by the loading level nor by the nanofiller type or concentration.

The potential influence of nanoparticle surface treatment or coupling agents on the optical properties and color stability of 3D printed provisional resins is an important consideration in understanding the overall performance of these materials [36,41]. Surface treatment, such as silane coupling agents, can improve the adhesion between nanoparticles and the resin matrix, leading to better dispersion and reduced agglomeration of nanoparticles [36]. This improved dispersion can result in more uniform optical properties and color distribution within the resin, ultimately enhancing color stability [36,41,44]. Additionally, surface treatment can also modify the surface chemistry of nanoparticles, influencing their interaction with light and thus affecting the overall optical properties of the resin. Moreover, the choice of coupling agent or surface treatment can affect the degree of light scattering and absorption within the resin matrix, which, in turn, influences its translucency and color appearance [36,37]. For example, certain surface treatments may enhance light transmission through the resin, resulting in a more translucent material with improved color stability, while others may reduce light transmission and increase opacity, leading to color changes [36]. Studies have shown that the selection of appropriate surface treatments or coupling agents is critical in optimizing the optical properties and color stability of nanoparticle-modified resins [37]. For instance, silane coupling agents have been found to effectively improve the dispersion of nanoparticles within resin matrices, resulting in enhanced color stability and translucency [36,41]. Similarly, other surface treatments, such as plasma treatment or functionalization, have been shown to positively impact the optical properties of nanoparticle-modified resins [36,43]. In summary, surface treatment or coupling agents play a significant role in determining the optical properties and color stability of nanoparticle-modified resins. By carefully selecting and optimizing these surface treatments, researchers can enhance the overall performance and esthetic qualities of 3D printed provisional restorations [37].

The selection of color measurement method, namely ΔE_00_, stems from the need to comprehensively evaluate color changes in dental resins modified with ZrO_2_NPs and SiO_2_NPs [25,33]. ΔE_ab_, representing the traditional CIELAB color difference formula, is widely used in dentistry and provides a straightforward and practical assessment based on perceptual color differences [43,49]. However, the accuracy of color assessment is improved by the use of the ΔE_00_ formula that includes adjustments for perceptual uniformity [43,49]. This method approximates the human eye’s nonlinear sensitivity to color differences, providing a more accurate representation of perceived color changes [45,46]. Therefore, using the ΔE_00_ method offers a robust analysis, accounting for contemporary approaches to color measurement in dental materials [45,48] and those selected in the present study.

ZrO_2_NPs showed a statistically significant color change with both concentrations. These results match those of a previous report which concluded that ZrO_2_NPs addition reduced the material’s translucency in proportion to its concentrations [50]. Aszrin et al. [51] indicated that the combination of varying concentrations of ZrO_2_, AlO_2_, and SiO_2_ fillers had an unanticipated negative affect on translucency. Variations in the optical properties of ZrO_2_NPs and their dispersion in the matrix could be the source of decreased translucency [50,51]. The opacity and crystallinity of these nanofillers is thought to be the cause of light diffusion impedance [43,45]. Additionally, ZrO_2_NPs cluster formations can reduce translucency by obstructing light transmission [51].

The addition of SiO_2_NPs showed a statistically insignificant change in color in both concentrations, with a greater color change at 1% reinforcement in both materials. However, all values for color change in both materials were clinically insignificant. A previous study has demonstrated that NPs had a minor effect on the optical properties of the auto-polymerizing provisional resin with no clinical perceptibility, with values ranging between 2.7 and 3.3 in darker shades. The A3 shade, on the other hand, expressed an acceptability threshold value of 1.8 with 0.25 wt% SiO_2_NPs [15]. As this study only tested A1 shade, both concentrations of SiO_2_NPs did not affect the optical properties. The ability to transmit light through the resin was not significantly reduced with these NPs [15,41,43]. The additive agent concentration, shape, size, and distribution in the mixture, all directly correlate with light scattering [15,41,43]. One likely explanation is the variation in refraction index between the different materials, which does not diffuse transmitted light equally. ZrO_2_NPs with a tetragonal shape had a significant impact on the color of the modified specimens in this investigation. According to Arikawa et al. [52], materials with small irregular fillers showed a sharper peak of light diffusion than those with large spherical fillers. The materials with the least amount of light scattering within the matrix and the highest degree of light transmission are those with the smallest-size reinforcing agent [53]. For the materials under evaluation, this could account for the varying degrees of translucency alterations brought on by the different NPs [52,53,54,55,56].

According to a previous report, clinical perceptibility and acceptability thresholds, this study revealed that the color variations in the ZrO_2_NPs-reinforced-resin were substantially greater than the average for ΔE_00_ for SiO_2_NPs. In both resins, ASIGA and NextDent, 0.5% ZrO_2_NPs resulted in ΔE_00_ values of 4.1 and 4.3, respectively, while 1% ZrO_2_NPs produced ΔE_00_ values of 5.8 and 5.2, respectively. In contrast, SiO_2_NPs in both concentrations presented the lowest color change perceptibility. These results are aligned with prior investigations that assessed the ΔΕ_00_ values after the addition of NPs [43,51].

The color change per NPs’ addition exhibited both high and low variations in the modified resins. Notably, a comparison between the two types of NPs indicated that ZrO_2_NPs induced more pronounced color changes than SiO_2_NPs. A closer examination of physical images revealed the emergence of a whitish color associated with ZrO_2_NPs, contributing to the overall color alterations in the provisional resins. These findings are supported by the ZrO_2_NPs-reinforced groups having ΔE_00_ values above the perceptible threshold compared to the SiO_2_NPs with values at the limit of the threshold. Among the other factors that affect the color is the loading ratio of NPs, which had a significant effect on the degree of color change (*p* = 0.002). Another element is the light absorption and scattering off the outer layer of the prosthesis and from within [53]. Translucency and perception of color are closely linked to light scattering [55,56]. Printed samples typically consist of multiple layers, each of which may have a different refractive index [53,55]. Light is reflected and transmitted by each layer and interface that make up the sample. The layers may reflect or transmit light at their interfaces, or scatter and/or absorb it within the layers [51,53,55]. Translucency variations from the same resin may be based on printing angle, different orientation of layers, scattering or absorption values and different overall measures from the contact points in the layers [53].

Due to the fact that discs from different brands vary in strength, this study’s findings advise clinicians that understanding the benefits, limitations, and quirks of the many provisional restoration fabrication procedures is essential to achieving these outcomes and preventing failures and difficulties. Test results for the tested NPs are satisfactory, and they can be recommended for long-term provisionalization in clinical practice. This might hold true for specimens that were disc-shaped printed provisional restorations, such as bridges and single units. As a result, caution should be taken when interpreting the results of this study to implement in real life until more research is undertaken using actual tooth-shaped temporary dental prostheses. This study emphasizes the importance of balance between strength, surface properties, and esthetics when selecting NPs for provisional resin reinforcement. It enables healthcare providers to draw comparisons between various systems in terms of how effectively the examined materials function under uniform settings. Because of their improved mechanical qualities, ease of handling, and consistency in results, new generations of these materials are expected to become more practical. When esthetics are the concern, clinicians are advised to use SiO_2_NPs more than ZrO_2_NPs due to their clinical color acceptability thresholds and enhanced patient satisfaction and acceptance. Both are recommended in esthetically undemanding areas like posterior provisional restorations. In addition, the modified provisional restoration has advantages over the pure group as it showed improvements in the properties that will result in better practical outcomes and patient satisfaction. The use of two different resin materials and two nanoparticles makes up for the strength of this study.

The limitations of this in vitro study include using disc-shape specimens that are different in configuration than the actual prostheses. In addition, the absence of simulated oral environment, such as saliva, bacteria, and masticatory load may affect the interpretation of this study’s results. Future investigations of provisional restoration made using other printing technologies in addition to aging processes such as thermal cycling and cyclic loading are required to mimic intraoral conditions. It is advised in future studies to analyze the effects on various aging processes, upon different printing technologies, to assess the long-term performance of the materials and different thermal, tribological properties’ analysis, as well as to compare alternate types of NPs or combinations of them, on different additional analysis techniques.

## 5. Conclusions

The incorporation of ZrO_2_NPs and SiO_2_NPs in 3D printed provisional resins increased hardness, while surface roughness was not affected. Regardless of the 3D printed provisional material type, ZrO_2_NPs’ addition produced noticeable color change, while 0.5% SiO_2_NPs showed small color changes not exceeding perceptibility thresholds. Consequently, care must be taken to choose a concentration that will improve the materials’ mechanical qualities without detracting from their esthetic value.

## Figures and Tables

**Table 1:** Nanoparticles details.

Nanoparticles	Manufacturer	Description	Surface Treatment	Mixing and Addition
ZrO_2_NPs	Shanghai Richem International Co., Ltd., Shanghai, China	0.5% and 1% weight spherical, white, and tetragonal particles (12 ± 3 nm; purity > 99%)	Silane coupling agent (3-(trimethoxysilyl) propyl methacrylate); Shanghai Richem International Co., Ltd., Shanghai, China	NPs powder was meticulously stirred with resin fluid for 30 min to ensure homogeneity
SiO_2_NPs	AEROSIL R812; Evonik-Degussa, Essen, Germany			

**Table 2:** Printing details.

Material	Manufacturer	
NextDent/Printer	NextDent (C&B NextDent, Shade N1, Soesterberg, The Netherlands)	
	NextDent 5100 3D printer (3D Systems, Rock Hill, SC, USA)	
ASIGA/Printer	ASIGA (Asiga DentaTOOTH, Shade A1, ASIGA, Erfurt, Germany)	
	ASIGA MAX printer (Asiga, Alexandria, NSW, Australia)	
Printing parameters	printing layer thickness	50 µm
	printing orientation	90 degrees
	post-curing time	30 min at 80 °C

**Table 3:** Three-way ANOVA for the interaction effects of the factors on the hardness and roughness.

Tested Properties	Interaction	Type III Sum of Squares	df	Mean Square	F	*p*
Hardness	Intercept	65,026.748	1	65,026.748	4012.617	0.000 *
	NP × concentration	57.562	1	57.562	3.552	0.064
	NP × material	22.770	1	22.770	1.405	0.240
	concentration × material	3.630	1	3.630	0.224	0.637
	NP × concentration × material	5.222	1	5.222	0.322	0.572
	Error	1166.801	72	16.206		
	Total	66,307.356	80			
Surface roughness Ra (µm)	Intercept	7.776	1	7.776	2562.904	0.000 *
	NP × concentration	0.002	1	0.002	0.601	0.441
	NP × material	0.013	1	0.013	4.153	0.045 *
	concentration × material	0.002	1	0.002	0.798	0.375
	NP × concentration × material	0.000	1	0.000	0.063	0.802
	Error	0.218	72	0.003		
	Total	8.114	80			
ΔE_00_	Intercept	1012.180	1	1012.180	3196.520	0.000 *
	NP × concentration	3.297	1	3.297	10.411	0.002 *
	NP × material	6.028	1	6.028	19.037	0.000 *
	concentration × material	0.002	1	0.002	0.005	0.943
	NP × concentration × material	1.152	1	1.152	3.638	0.060
	Error	22.799	72	0.317		
	Total	1296.009	80			

* statistically significant at 0.05 level of significance.

**Table 4:** Effect of different concentration levels of nanoparticles on the hardness and roughness and color changes of provisional resins.

**Tested Properties**	**NP**	**Concentration**	**ASIGA**	**NextDent**	***p*** **-Value**
Hardness (VHN)	ZrO_2_NP	Pure	22.3 (3.2)	23.4 (0.9)	0.621
		0.5%	29.59 (9.1) ^a^	26.78 (2.7) ^a^	0.073
		1%	29.67 (2.3) ^a^	28.74 (1.4) ^a^	0.077
		*p*-value	0.01 *	0.000 *	
	SiO_2_NP	Pure	22.3 (3.2)	23.4 (0.9)	0.621
		0.5%	29.33 (0.7) ^a^	29.69 (2.6) ^a^	0.531
		1%	27.0 (1.6) ^a^	27.23 (4.8) ^a^	0.334
		*p*-value	<0.001 *	0.001 *	
Surface roughness (Ra, µm)	ZrO_2_NP	Pure	0.26 (0.04)	0.33 (0.08)	0.032 *
		0.5%	0.23 (0.03)	0.33 (0.05)	0.001 *
		1%	0.28 (0.07)	0.36 (0.04)	0.042 *
		*p*-value	0.119	0.395	
	SiO_2_NP	Pure	0.26 (0.04)	0.33 (0.08)	0.032 *
		0.5%	0.28 (0.04)	0.33 (0.03)	0.044 *
		1%	0.32 (0.09)	0.34 (0.05)	0.472
		*p*-value	0.058	0.92	
Color changes ∆E_00_	ZrO_2_NP	0.5%	4.1 (0.4)	4.3 (0.3)	0.941
		1%	5.8 (0.4)	5.2 (0.4)	0.429
		*p*-value	0.000 *	0.000 *	
	SiO_2_NP	0.5%	1.01 (0.4)	1.51 (0.5)	0.040 *
		1%	1.14 (0.2)	1.85 (0.1)	0.038 *
		*p*-value	0.430	0.051	

* statistically significant at 0.05 level of significance. Same small letters in each column showed statistical insignificance between the pairs.

## Data Availability

The data are available upon request via email or phone to the corresponding author.
